# Housing Instability and Depression among US Mothers Following a Nonmarital Birth

**DOI:** 10.3390/ijerph181910322

**Published:** 2021-09-30

**Authors:** Sehun Oh, Ian Zapcic, Michael G. Vaughn, Christopher P. Salas-Wright, Yeonwoo Kim

**Affiliations:** 1 College of Social Work, The Ohio State University, Columbus, OH 43210, USA; zapcic.1@osu.edu; 2 College of Public Health and Social Justice, Saint Louis University, St. Louis, MO 63103, USA; michael.vaughn@slu.edu; 3 Graduate School of Social Welfare, Yonsei University, Seoul 03722, Korea; 4 School of Social Work, Boston College, Chestnut Hill, MA 02467, USA; Christopher.Salas-Wright@bc.edu; 5 Department of Kinesiology, University of Texas at Arlington, Arlington, TX 76010, USA; yeonwoo.kim@uta.edu

**Keywords:** depression, housing instability, motherhood, nonmarital birth

## Abstract

Mothers who had a nonmarital birth experience multiple risk factors for depression, including housing instability. Yet, important questions remain about the extent of long-term housing instability and its association with future depression among at-risk mothers. Using the Fragile Families and Child Wellbeing Study data, we examine cumulative housing instability over a 15-year period following nonmarital birth and its association with maternal depression. Based on a sample of 2279 mothers who had a nonmarital birth in 20 major US cities between 1998–2000, we examined their 15-year residential moves and housing arrangements. Then, we tested the associations between the cumulative residential moves and major depressive episodes (MDE) in Year 15 using logistic regression analysis. One in every four mothers had six or more residential moves in 15 years following a nonmarital birth. For each additional move, mothers reported up to 27.9% higher odds of having a past-year MDE in Year 15, translating into the prevalence increases from 6.0% (zero move) to 20.6% (10 moves). Our findings suggest that greater attention should be paid to housing needs among mothers following a nonmarital birth, including temporary housing assistance and more fundamental programs to reduce housing instability as preventive mental health services.

## 1. Introduction

US Mothers who give birth outside of marriage, disproportionately represented by Black/Hispanic residents in urban areas, are exceedingly vulnerable for depression due to their elevated risks of multiple adverse life conditions [1,2,3]. For instance, unmarried mothers at childbirth report a higher likelihood of living in poverty (33.7% with cohabiting partners and 52.9% without cohabiting partners) than their married counterparts, 12.3% [4]. Further, a majority of these mothers continue to head their families while experiencing relational instability. Even among those who were cohabiting at the time of childbirth, 60% of these relationships end within five years [5]. In addition, they often experience chronic and acute life stressors, such as precarious employment, greater job strain, and parenting stress [6]. Having their early-life education and work experiences often interrupted by pregnancy and childcare, many of the mothers work in occupational sectors (e.g., service, sales, domestic, and childcare) which are unstable, low-paying, or have nonstandard work hours [7,8]. Their exposures to poverty and associated adverse life conditions place them at elevated risk for depression. Latest statistics suggest that over 10% of mothers living in poverty experience a major depressive episode each year while fewer than 7% of mothers with household income above the poverty line did [9]. As unmarried mothers are currently responsible for 40 percent of all childbirths in the United States (US), depression continues to be a significant public health concern in the US.

One critical factor impacting depression among at-risk mothers requiring additional research attention is housing instability (defined in this study primarily as frequent residential moves) [6,10,11,12]. US-based research indicates that low-income unmarried mothers residing in urban areas experience frequent residential moves largely due to push mechanisms such as forced moves (e.g., evictions, fires, foreclosures) and relational instability, increasing stress levels while disrupting social ties [13]. Similar phenomena have been reported in other higher-income countries including the UK, where housing payment problems had detrimental effects on mental well-being among female heads of households [14]. Mothers in these adverse social contexts have limited opportunities to create a defined physical place where the stability provided by individual and family processes, eliciting feelings of safety, ownership, and empowerment as well as shaping social and cultural identity, take place [15]. In addition, housing instability is associated with a variety of infectious diseases and physical health problems such as obesity, hypertension, and diabetes as well as increased hospital visits, which may worsen mental health on their own or via potential costs and job losses [16,17,18]. Worse, the mental health symptoms experienced during housing instability may persist even after stable housing is obtained [19].

A variety of publicly funded programs exist in the US to assist with housing instability among disadvantaged individuals and families. However, evidence suggests that these large-scale programs do not provide sufficient long-term housing stability for at-risk mothers. For instance, public housing is intended to provide a temporary housing arrangement while the negatives associated with public housing, such as concentrated poverty and crime or deteriorating infrastructure, raise concerns [20]. Moreover, affordable housing and voucher programs often exhibit long waiting periods [21], making them less than ideal solutions for families experiencing sudden or frequent housing instability. The Section 8 voucher program, a means-tested subsidy program under which eligible recipients receive a voucher to cover a portion of the monthly rent for units, requires recipients to enter the general rental market, and participants have reported feeling stigmatized or overlooked by potential landlords [22]. Additionally, renters on assistance continue to experience increased financial burdens for rent and utilities [23].

Though existing literature has examined the relationships between housing instability and mental health disorders, there are also opportunities for further investigation. First, we need evidence to elucidate long-term housing instability among mothers following their nonmarital birth. As studies have indicated that unmarried mothers compared to their married counterparts are two-to-three times more likely to experience housing instability within the five years following childbirths [24], it is important to understand whether their housing situations persist over time. Second, housing instability needs to be examined as a determinant of maternal depression, especially among mothers who had a nonmarital birth. Prior studies primarily have examined maternal depression as a risk factor for homelessness [25]. Other studies focused on depression among the general population of female or mothers, mostly based on information either collected for a short period or via a retrospective self-report [26,27], or other at-risk populations such as children [28]. Lastly, there is a need for research that utilizes measurement and analysis specifically directed toward how housing instability cumulatively impacts maternal depression for mothers reaching middle age. An examination of cumulative housing instability may shed light on the elevated depression risk among mothers after a nonmarital birth, particularly those with children approaching adolescence or entering middle age [29].

### 1.1. Theoretical Background

Family stress model and life course theory collectively offer a useful framework to guide our investigation of housing instability as a key etiology of maternal depression. The family stress model [30,31] focuses on the economic dimension of socioeconomic status to examine its impact on family well-being. Conger and his colleagues [30] suggest that adverse economic conditions, such as low income, housing instability, and other negative financial events, increase family economic pressure. As a result, parents may experience impaired mental (e.g., depression) and other behavioral health (e.g., substance use disorders) [32]. Especially for families with children, poor mental and behavioral health among parents tend to increase conflicts with other family members including other parents/partners and children, adversely affecting their mental and behavioral well-being further [30,31]. Thus, when exposed to housing instability—often coupled with other adverse life conditions, unmarried mothers at childbirth are likely to face economic pressure, which deteriorates their mental health.

On the other hand, life course theory [33] can help us understand how housing instability may persist over time among families experiencing economic disadvantages. Life course theory posits that life domains are interconnected over time, generating the link between early life experiences and outcomes and those in later life [34]. Using the concept of cumulative advantage and disadvantage, it explains that early life disadvantages, such as economic hardship and single parenthood, may accumulate over the life course, widening intracohort disparities (i.e., married vs unmarried mothers in this example) [35,36,37]. At the same time, the interconnectedness exists across life domains, which allows one life domain’s condition to “spill over” into other life domain [38,39]. This implies that past or present housing instability is not only helpful to predict future housing instability, but also predicting challenges in other life domains, such as mental health in later life, by draining out psychological resources.

Despite the theoretical support for the relationship between cumulative housing instability and maternal depression, it remains as an empirical question. Specifically, it is not clear whether early life housing instability among mothers with a nonmarital childbirth will persist over time and how the instability will be associated with their housing arrangement patterns. More importantly, despite some evidence that link socioeconomic status and behavioral health [40,41], the associations between cumulated housing instability and future maternal depression among the at-risk mothers require empirical investigation in a longitudinal study design.

### 1.2. The Present Study

The present study aims to examine cumulative housing instability risks over a 15-year period and their associations with depression among mothers after a nonmarital birth. Using the data from the Fragile Families and Child Wellbeing Study (FFCWS), a population-based, longitudinal study of mothers who gave a birth outside of marriage, we examine the 15-year cumulative residential moves among the unmarried mothers since their childbirth. To provide salient housing contexts, housing arrangements from during the study period were also examined. We also test the effects of cumulative residential moves on maternal depression in Year 15 while controlling for major sociodemographic, health, and parenting stress. We expect that higher cumulative residential moves are associated with increased depression risk at Year 15 among mothers who had a nonmarital birth.

## 2. Materials and Methods

### 2.1. Data and Sample

Data were derived from the FFCWS, a longitudinal cohort study of 4898 children born in the United States between 1998 and 2000. Using a stratified random sampling design, mothers who gave birth were randomly selected from 75 hospitals within 20 U.S. cities with populations of 200,000 or more. Based on an oversampled nonmarital birth (roughly three quarters of the total focal children), FFCWS provides population estimates of an array of contextual factors related to parents and home/school/neighborhood environments as well as cognitive, emotional, physical, and behavioral child development outcomes. In the time since both mothers and fathers completed face-to-face interviews in the hospitals, a total of five follow-up interviews were conducted at children’s ages one (1999–2001), three (2001–2003), five (2003–2006), nine (2007–2010), and fifteen (2014–2017). Of 3709 unmarried mothers interviewed at baseline, 2349 mothers were primary caregivers of the focal children and thus interviewed at Year 15, indicating a retention rate of 63.3%. After dropping 70 cases (3.0%) with incomplete responses on depression and control variables at Year 15, our analytic sample included 2279 mothers who had a nonmarital birth in 1998−2000. For comparison purposes, 773 mothers married at childbirth were also examined. More details about the study design and sampling procedure of the FFCWS are available elsewhere [42]. By relying on de-identified public-use FFCWS data, this study is not considered human subjects research by the corresponding author’s research ethics committee.

### 2.2. Measures

#### 2.2.1. Major Depressive Episode (MDE)

We used a dichotomous measure of one or more MDE in the past year (0 = no episodes, 1 = one or more episodes) based on respondents’ self-reports to 15 items from the Composite International Diagnostic Interview—Short Form, Section A [43]. Consistent with the Diagnostic and Statistical Manual of Mental Disorders, Fourth Edition (DSM-IV), a respondent is considered to meet criteria for a past-year MDE if they report having two weeks of dysphoric mood or anhedonia lasting at least “most of the day”, and at least four out of seven function-related symptoms (e.g., trouble with sleep, trouble concentrating, thoughts about death) in the same 2-week period during the past year.

#### 2.2.2. Cumulative Residential Moves

During each follow-up interview from Years 1 to 15, respondents were asked “How many times have you moved since (the interview date of previous survey)?” Based on their self-reports from each follow-up interview, a total number of residential moves during the 15-year study period were computed by summing across the number of moves from the follow-up interviews.

#### 2.2.3. Housing Arrangements

Respondents were asked about their current housing situation at every follow-up interview from Year 1 to Year 15. The response categories included owning their home, renting their own apartment or house, living with family or friends who rent (with and without contributing part of the rent), and other (temporary housing or group shelter, mobile home, motel, public housing, and jail/prison).

#### 2.2.4. Controls

Sociodemographic characteristics at baseline included age at childbirth (1 = ages 15–19, 2 = ages 20–29, 3 = ages 30+), race/ethnicity (1 = White, 2 = Black, 3 = Hispanic, 4 = Other), educational attainment (1 = high school or lower, 2 = some or technical college, 3 = college or graduate degree), born in the U.S. (0 = no, 1 = yes), annual household income to poverty ratio (1 = 0–99%, 2 = 100–199%, 3 = 200%+), past-year Temporary Assistance for Needy Families [TANF] or Supplemental Nutrition Assistance Program [SNAP] assistance receipt status (0 = no, 1 = yes), and past-year government housing assistance receipt status (0 = no, 1 = yes). Year 15 control variables for main analyses included marital/cohabitation status (1 = married or cohabiting with the focal child’s other biological parent, 2 = married or cohabiting with someone else, 3 = not married or cohabiting) and number of children in household in addition to the aforementioned sociodemographic characteristics measured again at Year 15 (i.e., educational attainment, household income to poverty ratio, past-year TANF or SNAP receipt, past-year government housing assistance receipt). Moreover, to control for major stressors among the mothers who are more likely to be the sole caregivers, parenting stress and serious health conditions at Year 15 were also included. Parenting stress was measured by the 4-item scale (Cronbach’s alpha = 0.68) from the Panel Study of Income Dynamics based on four possible responses (1 = strongly agree, 2 = somewhat agree, 3 = somewhat disagree, 4 = strongly disagree). The scale included items, such as “I find that taking care of my child(ren) is much more work than pleasure,” and “I often feel tired, worn out, or exhausted from raising a family.” After reverse-coding, the responses were averaged to create an index of parenting stress, ranging between 1 and 4 (with 1 being the least and 4 being the highest level of stress). For serious health conditions, mothers who reported having diabetes, asthma, heart disease, seizures/epilepsy, and back problems that limit the amount or kind of work they do were coded 1, and 0 otherwise.

### 2.3. Statistical Analysis

Statistical analyses were conducted in four steps. First, we examined the baseline sociodemographic characteristics of mothers who were unmarried at the time of childbirth in comparison to their married counterparts. Second, we estimated the prevalence rates of cumulative residential moves during the first 15 years following a nonmarital birth. Third, we examined the proportions of respondents for each housing arrangement from Year 1 to Year 15. Lastly, a series of logistic regression analyses were conducted to test the relationship between cumulative residential moves for 15 years following the childbirth and past-year MDE in Year 15 among the mothers who had a nonmarital birth: an unadjusted model with residential moves and its squared term (Model 1: “The baseline model”), a model with residential moves adjusted for the full list of covariates including sociodemographic and health characteristics (Model 2: “The full-covariate model”), and a model with residential moves that retains only significant covariates from the full-covariate model (Model 3: “The simplified model”). To examine the unique depression risks associated with cumulative residential moves, predicted probabilities of having a past-year MDE were estimated based on the estimates obtained from the three logistic regression models. All estimates were weighted to account for the FFCWS’s sampling design using Stata 15.1 (StataCorp. LP, College Station, TX, USA).

## 3. Results

### 3.1. Sociodemographic Characteristics of Mothers Unmarried at Childbirth

Table 1 presents the baseline sociodemographic characteristics of mothers who were unmarried at the time of childbirth in comparison to their married counterparts. At baseline, the majority of unmarried mothers were ages 20–29 (55.7%, 95% CI = 50.5–60.7%), Black (55.4%, 95% CI = 50.5–60.2%), completed high school or lower (80.1%, 95% CI = 76.3–83.5%), and lived in poverty (52.0%, 95% CI = 46.9–57.1%). In terms of government assistance, 38.2% (95% CI = 33.9–42.8%) of them received TANF or SNAP benefits and 24.5% (95% CI = 20.4–29.1%) received housing assistance in the past year. Compared to the married group, unmarried mothers were more likely to be teens at childbirth (AOR = 4.98, 95% CI = 2.19–11.35); be Black (AOR = 8.52, 95% CI = 4.46–16.31), Hispanic (AOR = 3.45, 95% CI = 1.66–7.17) or other racial/ethnic group (AOR = 4.30, 95% CI = 1.46–12.70); have completed high school or lower (AOR = 15.70, 95% CI = 7.24–34.07); and have lived in poverty (AOR = 2.69, 95% CI = 1.38–5.27).

### 3.2. Cumulative Residential Moves and Housing Arrangements Following a Nonmarital Birth

**Figure 1:**
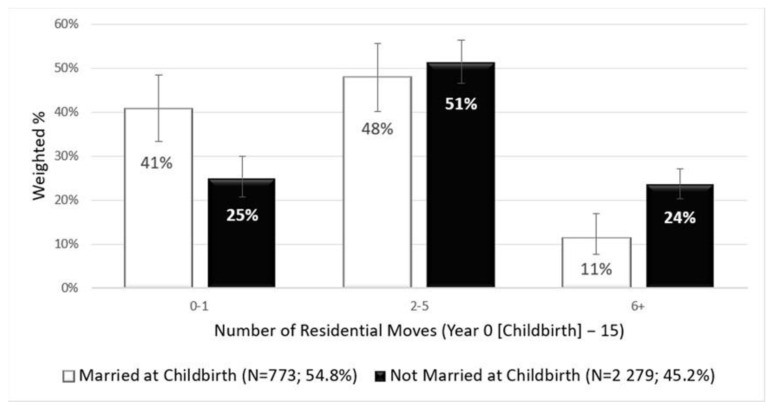
Number of 15–year cumulative residential moves among U.S. mothers by marital status at childbirth, Fragile Families and Child Wellbeing Study (*n* = 3052).

Figure 1 displays the proportions of US mothers who moved 0–1, 2–5, and 6+ times during the first 15 years following a nonmarital birth. About 24% (95% CI = 22.0–28.9%) of the mothers unmarried at childbirth reported having moved six or more times before their children turned 15 years old, which was significantly higher than the rate among their married counterparts (11% [95% CI = 7.6–14.9%]). On the other hand, 41% (95% CI = 34.6–48.2%) of the mothers married at childbirth have not moved at all or once for the 15 years, while only 25% (95% CI = 20.0–28.7%) of the mothers unmarried at childbirth belonged to this category. A supplementary analysis (see Table A1) indicates that mothers reporting six or more moves during the study period are likely to experience other socioeconomic economic disadvantages, such as having a nonmarital childbirth while teenaged (24.3%), educational attainment of high school or lower (70.9%) and living in poverty at Year 15 (45.1%).

Table 2 shows housing arrangements at each follow-up interview from Year 1 to Year 15. While the mothers who had a nonmarital birth either owned or rented an apartment/house more gradually over time, only 15.6% (95% CI = 13.0–18.7%) were able to own a house in Year 15. Instead, over 70% of them were renters, while 9.9% (95% CI = 7.7–12.7%) of them continued to live with family or friends in Year 15. On the other hand, Table A2 shows that the percentages of the married group renting an apartment/house have gradually decreased from 48.2% (95% CI = 40.1–56.4%) in Year 1 to 39.0% (95% CI = 31.1–47.6%) in Year 15. The decreases in renters among the mothers married at childbirth were offset by the increases in homeownership from 43.1% (95% CI = 35.7–50.9%) in Year 1 to 56.0% (95% CI = 47.8–63.9%) in Year 15.

### 3.3. Effects of Cumulative Residential Moves on MDE among Mothers after a Nonmarital Birth

**Figure 2:**
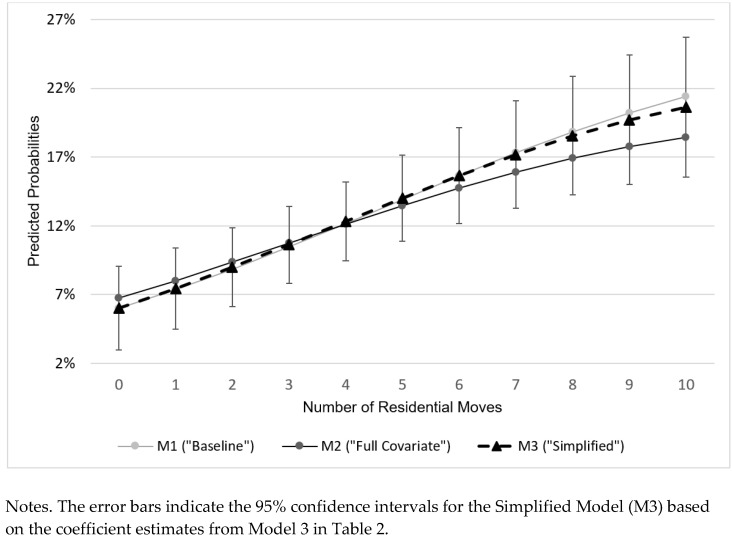
Predicted probabilities of past-year major depressive episodes among US mothers by number of residential moves during the first 15 years after a nonmarital birth, Fragile Families and Child Wellbeing Study (*n* = 2279).

Table 3 shows the effects of cumulative residential moves on MDE among the mothers in Year 15 following a nonmarital birth. More residential moves predicted higher odds of a past-year MDE. The three models with different sets of sociodemographic and health covariates suggest between 23.6% (95% CI = 1.071–1.427; the Full-Covariate Model [M2]) and 27.9% (95% CI = 1.102–1.484; the Simplified Model [M3]) higher odds of having a MDE in the past year for each additional residential move. Based on the estimates, Figure 2 displays the predicted probabilities of having a MDE in Year 15 for each number of cumulative residential moves since a nonmarital birth. The simplified model (M3) suggests that 6.0% (95% CI = 3.0–9.1%) of the unmarried mothers who have not moved at all during the study period were anticipated to have a MDE in Year 15. The likelihood of having a MDE was more than three times higher for those who moved 10 times in 15 years, indicating the rate of 20.6% (95% CI = 15.6–25.7%).

## 4. Discussion

The present study suggests that mothers who were unmarried at the time of childbirth are experiencing disproportionate risks of cumulative residential moves over time. Nearly a quarter of the at-risk mothers have moved six or more times in 15 years following their childbirth while less than one half of their married counterparts did. Furthermore, only 25 percent of the mothers unmarried at childbirth have not moved at all or moved once during the same period while more than 40 percent of the married group was within that category. An examination of housing arrangement changes suggests that higher cumulative residential moves among the mothers following a nonmarital birth may be closely related to persistently low homeownership. After 15 years following their nonmarital births, only 15.6 percent of them owned a house. On the contrary, 56.0 percent of their married counterparts were homeowners in the same timeframe. Instead, the majority of the mothers unmarried at childbirth continue to be renters or “doubled-up” families (i.e., living with family, friends, or other non-relatives), whereas only 39.0 percent and 3.6 percent of the married counterparts were renters and “doubled-up” families in Year 15, respectively. As over 86% of the unmarried mother sample are represented by Black and Hispanic mothers, it is also important to note that the cumulative disadvantages would heavily fall on these racial/ethnic minority mothers and their family members, including children.

The present study’s findings showed that the disproportionate housing instability risks could consistently pose a great threat to unmarried mothers’ mental health in their later life. Each additional residential move among the mothers who had a nonmarital birth raised the odds of having a MDE in Year 15 by 23.6% or higher. When the number of residential moves accumulated to 10 times, the likelihood of having a MDE in Year 15 tripled from 6.0% to 20.6% when compared to mothers who have not moved at all. The strong impact of housing instability on maternal mental health is consistent with previous studies that examined various mother populations with disadvantages [44]. Considering multiple pathways that housing instability may adversely affect maternal mental health [45,46], a more comprehensive model which sheds light on the pathways needs to be further investigated to inform preventive efforts, aiming to reduce housing instability and co-occurring risks among at-risk mothers.

Policies and programs to assist housing among disadvantaged mothers can also be important service elements as preventive measures of maternal depression among unmarried mothers by addressing housing instability problems. Supplemental analyses (not shown) suggest that over 61% of mothers unmarried at childbirth (42.5% and 55.1% in the forms of public housing and the Housing Voucher, respectively) reported having received housing assistance at some point during the 15-year study period. However, it is still concerning that one fourth of the mothers who had a nonmarital birth have moved six or more times, despite the fact that over 60% of them have received housing assistance at least once during the 15-year time period. To maximize the gains from housing assistance in reducing long-term housing instability, further research is needed to better understand how the housing assistance programs may help to reduce overall housing instability, especially at the time of the greatest housing needs.

In addition to the temporary housing assistance, more fundamental programs that help disadvantaged mothers and their children increase their homeownership and financial capabilities are needed [47,48,49,50]. One example of such programs is Individual Development Accounts (IDA). In IDA programs, governments match funds to low-income recipients for their savings so that the participants may use the savings later for homeownership, education, and microenterprise, as well as offering homeownership and mortgage counseling to assist with the planning [47]. Despite the promise of IDA programs, preliminary results were somewhat mixed. After 10 years of the program, only IDA program participants whose baseline income levels were above the sample median showed increases in the rate and duration of homeownership, implying that the program was beneficial for more advantageous participants [48].

Several limitations of this study should be noted. First, self-reported depressive symptoms and residential moves may have been biased due to social desirability or recall bias. Second, attrition might have affected the effects of housing instability on maternal depression despite the acceptable retention rate of nearly 65% after 15 years from the baseline. Supplementary analyses (not shown) suggest that those who dropped out of the follow-up surveys were younger and had lower education, while no differences were found in the characteristics such as race/ethnicity, income to poverty, and welfare or housing assistance receipt at baseline. The higher likelihood of dropping out from surveys for more disadvantaged populations indicates that the effects of housing instability on MDE were likely to be underestimated. Third, the present study does not distinguish unmarried mothers by their partner status at childbirth, which may merit further investigation. About one half of unmarried mothers have cohabiting partners, who may be able to provide support and thus alleviate the mothers’ housing and mental health needs until the relationships last [5]. However, existing literature indicates that nearly 60% of those eventually end their relationships within five years, and thus the support may not last long [5]. In fact, our supplemental analysis suggests that there were only slight differences in the patterns of housing instability between unmarried mothers with and without cohabiting partners at childbirth. While the present study focused on unmarried mothers, future studies are recommended to inform the potential differences in housing and mental health needs by their partner status over time. Lastly, the present study does not test the mechanism regarding how housing instability directly or indirectly influences maternal depression via potential family or other environmental mediators. While key sociodemographic and health-related variables were included in our models, this study primarily focused on estimating the total effects of cumulative residential moves on maternal depression among at-risk mothers. Future studies are encouraged to incorporate key mediators to extend theoretical models such as the Family Stress Model [31].

## 5. Conclusions

The present study contributes to our understanding of depression by examining the cumulative effect of one of the major economic stressors among emerging at-risk mother populations in the US, i.e., housing instability. Due to their greater risk of family instability and limited economic resources, nearly one quarter of the mothers following a nonmarital birth reported having moved six or more times in 15 years, while only 15.66% of them became homeowners at the end of the study period. The greater housing needs among female populations are observed consistently across many higher income counties, but recent decreases in government spending on housing and more restrictive benefit rules in some counties such as UK raises major concerns for housing stability and mental health among women, especially at-risk mothers [51,52]. Given the strong and consistent effects of cumulative residential moves on maternal depression, it is important to consider temporary housing assistance programs as well as more fundamental programs to reduce housing instability such as homeownership or employment programs as a major preventive mental health service element for the at-risk mothers with housing needs. Additionally, future research is needed to better understand the complex mechanisms about how cumulative residential moves affect depression among the mothers in later life and to inform service provision effective in reducing the major mental health problems among the mothers following a nonmarital birth.

## Figures and Tables

**Table 1:** Baseline sociodemographic characteristics among U.S. mothers by marital status at childbirth in 1999−2001, Fragile Families and Child Wellbeing Study (*n* = 3052).

Sociodemographic Characteristics	Married (*n* = 773; 54.8%)		Not Married (*n* = 2279; 45.2%)		AOR	95% CI
	%	95% CI	%	95% CI		
Age						
15–19	2.5	1.4–4.4	24.1	19.8–29.0	4.98 ***	2.19−11.35
20−29	51.3	43.5–58.9	55.7	50.5–60.7	0.77	0.44−1.35
30+	46.3	38.7–54.0	20.2	16.0–25.2	1.00	-
Race/ethnicity						
White	44.7	37.2–52.5	11.0	8.4–14.4	1.00	-
Black	14.7	10.4–20.3	55.4	50.5–60.2	8.52 ***	4.46−16.31
Hispanic	30.2	23.2–38.4	31.3	27.0–36.0	3.45 **	1.66−7.17
Other	10.4	7.0–15.3	2.3	1.4–3.8	4.30 **	1.46−12.70
Education						
HS or lower	40.9	33.0–49.3	80.1	76.3–83.5	15.70 ***	7.24−34.07
Some college	21.4	16.8–26.9	18.3	15.0–22.2	8.16 ***	4.03−16.52
College degree	37.7	30.8–45.2	1.5	1.0–2.3	1.00	-
Income to poverty ratio						
0–99%	24.5	16.6–34.7	52.0	46.9–57.1	2.69 **	1.38−5.27
100–199%	15.5	11.5–20.7	28.6	24.0–33.6	2.64 **	1.46−4.78
200%+	60.0	51.2–68.1	19.4	15.6–23.8	1.00	-
Born in U.S.						
Yes	65.6	57.2–73.1	82.5	77.9–86.3	1.00	-
No	34.4	26.9–42.8	17.6	13.7–22.2	0.26 ***	0.13−0.50
TANF/SNAP receipt						
Yes	11.2	6.6–18.3	38.2	33.9–42.8	1.34	0.70−2.56
No	88.8	81.7–93.4	61.8	57.2–66.2	1.00	-
Housing assistance receipt						
Yes	8.8	4.8–15.8	24.5	20.4–29.1	1.17	0.52−2.62
No	91.2	84.2–95.2	75.6	71.0–79.6	1.00	-

Notes. TANF = Temporary Assistance for Needy Families. SNAP = Supplemental Nutrition Assistant Program. All percentages were weighted using sample weights. Adjusted odds ratios (AOR) were adjusted for baseline sociodemographic characteristics including age, race/ethnicity, educational attainment, annual household income, U.S. birth status, TANF/SNAP receipt status, and government housing assistance receipt status. ** *p* < *0*.01, *** *p* < 0.001.

**Table 2:** Weighted percentages of housing arrangements among US mothers following a nonmarital birth, Fragile Families and Child Wellbeing Study (*n* = 2279).

Housing Arrangement	Year 1	Year 3	Year 5	Year 9	Year 15
Own	6.2 (4.4–8.7)	7.9 (5.6–11.0)	10.2 (7.5–13.7)	14.3 (11.0–18.4)	15.6 (13.0–18.7)
Rent	61.2 (56.4–65.8)	67.1 (62.2–71.6)	69.5 (64.5–74.0)	70.2 (65.4–74.6)	71.5 (67.4–75.2)
Live with family or friends	30.5 (26.3–35.0)	23.5 (19.5–28.1)	19.4 (15.5–23.9)	14.7 (11.6–18.3)	9.9 (7.7–12.7)
Other	2.1 (1.0–4.1)	1.5 (0.9–2.5)	0.9 (0.5–1.6)	0.9 (0.6–1.3)	3.0 (1.8–5.1)

Note. Other includes temporary/group shelter, mobile home, motel, public housing, and jail/prison. The numbers in the parenthesis present the 95% confidence interval for each weighted percentage.

**Table 3:** The effects of 15-year cumulative residential moves on past-year major depressive episodes among US mothers following a nonmarital birth, Fragile Families and Child Wellbeing Study (*n* = 2279).

Variables	M1: Baseline Model		M2: Full Covariate Model		M3: Simplified Model	
	AOR	95% CI	AOR	95% CI	AOR	95% CI
**Key Covariates**						
No. of residential moves	1.255 **	1.072–1.470	1.236 **	1.071−1.427	1.279 **	1.102−1.484
No. of residential moves^2^	0.992 †	0.982–1.002	0.992 *	0.984−0.999	0.990 *	0.982−0.999
Parenting stress			2.253 ***	1.591−3.191	2.315 ***	1.625−3.298
Serious health conditions			3.091 **	1.448−6.598	3.314^**^	1.549−7.089
**Sociodemographic Controls**						
Age						
30–34			0.861	0.333−2.225		
35–44			0.791	0.346−1.808		
45+			1.000	-		
Race/ethnicity						
White			1.000	-		
Black			0.635	0.358−1.127		
Hispanic			1.336	0.591−3.020		
Other			0.710	0.231−2.179		
Born in U.S.						
No			0.344	0.084−1.403		
Yes			1.000	-		
Educational attainment						
High school or lower			1.304	0.662−2.566		
Some college			0.870	0.443−1.710		
College degree			1.000	-		
Marital/cohabitation status						
W/other biological parent			1.000	-		
W/someone else			1.143	0.542−2.410		
No			1.413	0.662−3.014		
No. of children in household			1.079	0.894−1.302		
Income to poverty ratio						
0–99%			1.059	0.426−2.635		
100–199%			0.661	0.299−1.461		
200%+			1.000	-		
Received TANF or SNAP						
No			1.000	-		
Yes			1.568	0.837−2.936		
Received housing assistance						
No			1.000	-		
Yes			0.585 †	0.330−1.037		

Notes. The Simplified Model (M3) retained only significant covariates from the Full Covariate Model (M2). Serious health conditions include diabetes, asthma, heart disease, seizures/epilepsy, and back problems that limit the amount or kind of work the mothers do. TANF = Temporary Assistance for Needy Families. SNAP = Supplemental Nutrition Assistant Program. † < 0.1, * *p* < *0*.05, ** *p* < *0*.01, *** *p* < *0*.001.

## Data Availability

The data presented in this study are available for access by the general public through the Inter-university Consortium for Political and Social Research (ICPSR) at the University of Michigan—Ann Arbor (https://www.icpsr.umich.edu/web/DSDR/studies/31622/summary [accessed on 28 January 2019]).

## Appendix A

**Table A1:** Socioeconomic characteristics among US mothers by cumulative residential moves from Year 1 to Year 15 following a childbirth, Fragile Families and Child Wellbeing Study (*n* = 3052).

Sociodemographic Characteristics	0–1 Move (*n* = 717; 23.5%)		2–5 Moves (*n* = 1473; 48.3%)		6 + Moves (*n* = 862; 28.2%)	
	%	95% CI	%	95% CI	%	95% CI
Age at nonmarital childbirth						
15–19	3.1	1.8–5.5	13.1	10.0–16.9	24.3	18.7–30.9
20−29	44.8	37.0–52.9	57.0	50.6–63.1	64.0	56.6–70.8
30+	52.1	44.1–59.9	30.0	24.2–36.5	11.7	7.4–17.9
Race/ethnicity						
White	40.0	32.2–48.4	29.4	23.4–36.4	16.7	12.1–22.5
Black	26.2	20.5–32.9	35.0	29.8–40.6	52.1	44.6–59.5
Hispanic	26.2	20.2–33.3	28.9	23.6–34.9	25.6	19.0–33.4
Other	7.5	4.2–13.0	6.7	4.1–10.7	5.7	2.4–12.8
Education						
HS or lower	46.2	38.4–54.3	57.7	51.4–63.8	70.9	63.4–77.4
Some college	20.6	15.7–26.6	24.7	19.9–30.2	17.4	13.3–22.5
College degree	33.2	26.3–40.8	17.6	12.9–23.6	11.7	6.6–19.9
Employment status						
Not employed	25.4	18.1–34.4	29.7	24.0–36.2	30.3	24.1–37.2
Employed	74.6	65.6–81.9	70.3	63.8–76.1	69.7	62.8–75.9
Income to poverty ratio (YR 15)						
0–99%	29.2	21.3–38.7	34.8	28.7–41.4	45.0	37.9–52.4
100–199%	15.8	11.3–21.5	23.7	19.0–29.1	29.6	22.9–37.4
200%+	55.0	46.5–63.2	41.5	35.2–48.2	25.4	18.7–33.5
Marital/cohabitation status (YR 15)						
W/other biological parent	67.1	59.9–73.7	42.4	36.1–48.9	21.6	15.9–28.6
W/someone else	6.5	4.1–10.0	21.6	16.8–27.4	29.5	23.5–36.4
No	26.4	20.4–33.4	36.0	30.6–41.8	48.9	41.5–56.3

**Table A2:** Weighted percentages of housing arrangements from Year 1 to Year 15 among US mothers married at childbirth, Fragile Families and Child Wellbeing Study (*n* = 773).

Housing Arrangement	Year 1	Year 3	Year 5	Year 9	Year 15
Own	43.1 (35.7–50.9)	47.2 (39.4–55.1)	50.8 (42.8–58.8)	54.6 (46.2–62.8)	56.0 (47.8–63.9)
Rent	48.2 (40.1–56.4)	44.4 (36.2–52.8)	43.5 (35.3–52.0)	41.7 (33.5–50.5)	39.0 (31.1–47.6)
Live with family or friends	7.9 (4.2–14.6)	8.0 (4.2–14.7)	5.4 (3.3–8.9)	3.7 (2.0–6.7)	3.6 (1.8–7.1)
Other	0.7 (0.2–2.4)	0.5 (0.2–1.1)	0.3 (0.1–1.1)	0.1 (0.1–0.1)	1.4 (0.5–3.9)

Note. Other includes temporary/group shelter, mobile home, motel, public housing, and jail/prison.
